# Ketamine Decreases Resting State Functional Network Connectivity in Healthy Subjects: Implications for Antidepressant Drug Action

**DOI:** 10.1371/journal.pone.0044799

**Published:** 2012-09-24

**Authors:** Milan Scheidegger, Martin Walter, Mick Lehmann, Coraline Metzger, Simone Grimm, Heinz Boeker, Peter Boesiger, Anke Henning, Erich Seifritz

**Affiliations:** 1 Institute for Biomedical Engineering, University and ETH Zurich, Zurich, Switzerland; 2 Clinic of Affective Disorders and General Psychiatry, Psychiatric University Hospital, Zurich, Switzerland; 3 Department of Psychiatry, Otto-von-Guericke University, Magdeburg, Germany; 4 Department of Behavioral Neurology, Leibniz Institute for Neurobiology, Magdeburg, Germany; 5 Clinical Affective Neuroimaging Laboratory (CANLAB), Center for Behavioral and Brain Sciences, CBBS, Magdeburg, Germany; 6 Cluster Languages of Emotion, Freie Universität Berlin, Berlin, Germany; 7 Department of Psychiatry, Charité, CBF, Berlin, Germany; 8 Zurich Center for Integrative Human Physiology (ZIHP), University of Zurich, Zurich, Switzerland; 9 Neuroscience Center Zurich, University of Zurich and ETH Zurich, Zurich, Switzerland; University G. D'Annunzio, Italy

## Abstract

Increasing preclinical and clinical evidence underscores the strong and rapid antidepressant properties of the glutamate-modulating NMDA receptor antagonist ketamine. Targeting the glutamatergic system might thus provide a novel molecular strategy for antidepressant treatment. Since glutamate is the most abundant and major excitatory neurotransmitter in the brain, pathophysiological changes in glutamatergic signaling are likely to affect neurobehavioral plasticity, information processing and large-scale changes in functional brain connectivity underlying certain symptoms of major depressive disorder. Using resting state functional magnetic resonance imaging (rsfMRI), the „dorsal nexus “(DN) was recently identified as a bilateral dorsal medial prefrontal cortex region showing dramatically increased depression-associated functional connectivity with large portions of a cognitive control network (CCN), the default mode network (DMN), and a rostral affective network (AN). Hence, Sheline and colleagues (2010) proposed that reducing increased connectivity of the DN might play a critical role in reducing depression symptomatology and thus represent a potential therapy target for affective disorders. Here, using a randomized, placebo-controlled, double-blind, crossover rsfMRI challenge in healthy subjects we demonstrate that ketamine decreases functional connectivity of the DMN to the DN and to the pregenual anterior cingulate (PACC) and medioprefrontal cortex (MPFC) via its representative hub, the posterior cingulate cortex (PCC). These findings in healthy subjects may serve as a model to elucidate potential biomechanisms that are addressed by successful treatment of major depression. This notion is further supported by the temporal overlap of our observation of subacute functional network modulation after 24 hours with the peak of efficacy following an intravenous ketamine administration in treatment-resistant depression.

## Introduction

Based on the increasing evidence of glutamate-modulating agents having strong and rapid antidepressant properties [1], [2], the NMDA receptor antagonist ketamine has been firmly established as a research tool for the investigation of the neurobiology of the glutamatergic system in major depressive disorder (MDD) and novel molecular targets associated with rapid onset of antidepressant drug action [3]–[7]. Although its exact mechanism of action is still unknown, various neuronal and molecular pathways have been investigated in animal models and are proposed to critically mediate its antidepressant effects [8]–[11]. Here, we aim to investigate pharmacological changes in functional connectivity in the healthy human brain as a model for ketamine's antidepressant action in order to elucidate its systems level biomechanisms. In the following sections, we briefly review glutamatergic mechanisms that are relevant to ketamine's drug action and that constitute a theoretical framework for the understanding of the neuronal adaptations that are accessible by pharmacological resting state functional magnetic resonance imaging (rsfMRI).

### 1. The glutamatergic system as a target for antidepressant intervention

In general, we hypothesize that the therapeutic potential of ketamine may be explained by reversing disturbances in the glutamatergic system [12] and thus restore parts of a disrupted neurobehavioral homeostasis in MDD, where several structural, metabolic, and functional abnormalities have been described previously [13]–[20]. Based on converging evidence from neuroimaging, neuropathological, and therapeutic intervention studies, the depressed state can be characterized by the tendency to enter and to remain in an inappropriate mode of information processing in limbic-cortico-striato-pallido-thalamic circuits that subserve the regulation of mood [21]–[23]. Since glutamate is the most abundant and major excitatory neurotransmitter in the human brain, pathophysiological changes in glutamatergic signaling associated with chronic stress exposure and disease progression emerge as a powerful explanatory framework to integrate the observed findings into a comprehensive disease model and provide novel molecular targets for therapeutic interventions [1]. This notion is further supported by several findings at different levels of neuronal organization, demonstrating beneficial effects of ketamine on glutamatergic signaling [24]–[28], AMPA-to-NMDA-receptor throughput [11], [29], intracellular signaling [8], [10], and neurotrophic factors [30], [31]. In conclusion, those findings indicate that ketamine may have a stimulating effect on overall glutamate-glutamine-cycling, which is supposed to be reduced in MDD [12], [17], [32].

Multimodal imaging studies combining MR spectroscopy and fMRI raised the interest for the investigation of the neurochemical basis of blood oxygen level-dependent (BOLD) signal fluctuations during activity [17], [33], [34] and at rest [35], [36]. Specifically in depressed patients, altered negative BOLD responses in the default mode network (DMN) [37] could be found during emotional processing tasks [38], [39], with decreased negative BOLD signal amplitudes being positively correlated with lower glutamate concentrations within the pregenual anterior cingulate cortex (PACC) [17]. A current investigation by Salvadore et al. (2009) importantly revealed magnetoencephalographic activation in the PACC to be predictive of subsequent treatment response to ketamine in MDD, thus providing a link between ketamine efficacy and glutamatergic dysfunction in the ACC [40]. Interestingly, altered resting state functional connectivities can be traced back to specific glutamatergic abnormalities within distinct neuronal networks in depressed patients as well [35]. In conclusion, glutamatergic signaling and brain energy metabolism seem to be altered in MDD and might be reflected in changes of functional signals up to the systems level and explain some crucial aspects of depressive symptomatology. Targeting the glutamatergic system by glutamate-modulating drugs such as ketamine might thus hold considerable promise for the development of new treatments for mood disorders.

### 2. Resting state functional connectivity as a biological marker for antidepressant intervention

Recent advances in resting state functional connectivity neuroimaging techniques suggest their utility for the investigation of (1) intrinsic brain connections in the healthy human brain, (2) pathophysiological alterations in disease states and (3) changes in neuronal network dynamics following therapeutic interventions [41], [42]. The characterization of changes in functional connectivity between brain networks subserving distinct psychophysiological functions might explain how various psychiatric symptoms arise from disrupted connectivities between distinct functional networks. Several dysfunctions in cortico-limbic neurocircuits [43], [44] as well as task-positive and task-negative systems [45] have been reported in previous studies of resting state functional connectivity in depressed patients. Recently, the „dorsal nexus” (DN) was defined as a bilateral dorsal medial prefrontal cortex (DMPFC) region showing dramatically increased depression-associated fMRI connectivity with large portions of the cognitive control network (CCN), the default mode network (DMN), and affective network (AN) [46]. Hence, reducing increased connectivity of the DN might play a critical role in reducing depressive symptomatology and thus represent a potential therapeutic target for affective disorders. The hypothesis that decreasing the activity of the DN might be a potential marker of antidepressant drug intervention was tested in a recent study in healthy subjects, showing reduced connectivity between the left DN seed region and the left hippocampus after selective serotonin reuptake inhibitor (SSRI) administration (citalopram, 20 mg, given daily for seven days) in a double-blind placebo-controlled design [47]. Interestingly, in another study the SSRI citalopram and the selective norepinephrine reuptake inhibitor (NARI) reboxetine reduced subcortical-cortical connectivities between the amygdala and the medial and orbitofrontal prefrontal cortices [48]. In conclusion, rsfMRI provides sufficient sensitivity and specificity for clinical applications including research studies focussing on disease biomarkers and pharmacological interventions. Recent evidence points to altered functional connectivity within and between critical neurocircuits in MDD. Reducing abnormally increased connectivities in those functional networks might represent a general response pattern to antidepressant drug treatment.

### 3. Investigating large-scale neural network dynamics following ketamine administration

**Figure 1:**
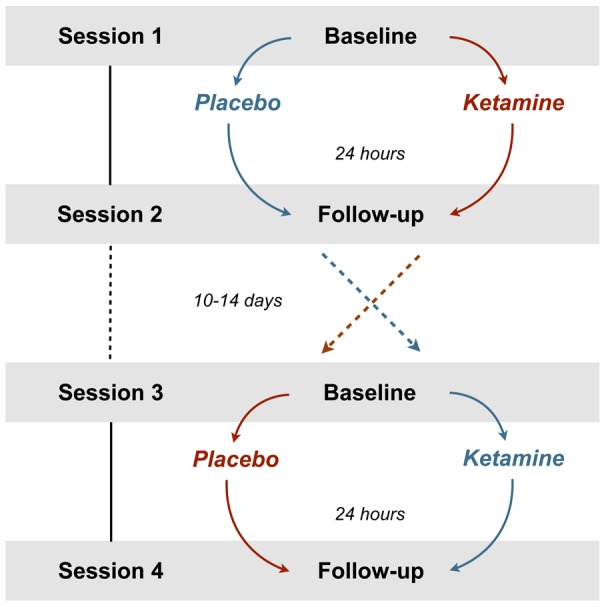
The randomized, double-blind, placebo controlled crossover design. Session 1 and 2 were completed by n = 19, session 3 and 4 by n = 17 (1 dropout per group). The blue and the red path indicate the randomly assigned order of administration.

Little is known about how ketamine affects large-scale neural network dynamics in the healthy human brain and whether it has the potential to restore the aberrant functional connectivities seen in MDD. Based on previous findings with other antidepressants, we hypothesized that ketamine at subanaesthetic doses will decrease the cortico-limbic resting state connectivity in healthy subjects as a general response pattern. In addition, we aimed to evaluate the pharmacological effects of ketamine on resting state connectivities via the DN [46] as a model for antidepressant drug intervention. To test our hypothesis, we examined 19 healthy subjects in a randomized, placebo-controlled, double-blind, crossover study. Every subject underwent four MR scan sessions including two baseline and two post infusion measurements 24 hours following a subanaesthetic intravenous dose of S-ketamine, or saline, respectively (Fig. 1). The 24 h follow-up interval was based on the evidence that ketamine decreases depressive symptomatology most effectively one day after a single intravenous infusion [4]. We thus aimed at directly probing the effect of a glutamatergic antidepressant drug on resting state functional connectivity in healthy subjects.

## Methods

### Ethics Statement

The study was approved by the University of Zurich institutional review board, and all subjects gave written informed consent before screening.

### Subjects

Healthy subjects (n = 19, mean age, 40.5+7.5 [standard deviation]; body mass index, 24+4.1; 9 males) without any psychiatric, neurological, or medical illness were self-referred from online study advertisements. All subjects underwent a psychiatric interview and medical examination. Only medication-free subjects that were healthy according to physical examination, electrocardiogram, blood and urine analyses were included in the study. Exclusion criteria were a history of psychiatric/neurological diseases, drug abuse, concurrent medication, cardiovascular disease, anaemia, thyroid disease, any somatic disease affecting drug metabolism and excretion (e.g. renal or liver disease), MR exclusion criteria, pregnancy, and left handedness.

### Study design

17 out of 19 subjects (one study dropout per ketamine or placebo run due to personal reasons) completed a total of four rsfMRI sessions in a double-blind, randomized, crossover study design (Fig. 1). The order of placebo and ketamine administration was assigned by an external third party. Participants were stratified for sex and age and randomly assigned to both groups (ketamine-placebo or placebo-ketamine) with a randomization ratio of 1∶1 to assertain matched groups. The baseline rsfMRI scan was followed by an intravenous (i.v.) infusion (45 mins) of either S-ketamine (0.25 mg/kg, Ketanest® S, Pfizer, Zurich, Switzerland) or saline (0.90% w/v of NaCl) outside the scanner. Previous clinical trials mostly used an i.v. dose of 0.5 mg/kg of racemic ketamine (R/S enantiomer ratio of 1∶1). The S(+)-isomer of ketamine is characterized by a 3–4 times higher affinity or potency at specific receptors, so that a dose reduction of 50% is recommended [49]. Since the antidepressant effect of ketamine is most prominent after one day [4], the follow-up fMRI scans were scheduled 24 hours after the ketamine or placebo infusion in order to assess the related effects on neuronal network dynamics that might contribute to the understanding of its antidepressant efficacy. To avoid possible carry-over effects, the time lag between the two baseline measurements was set to at least ten days. The time of day for all the imaging sessions was kept constant for every participant.

### Psychometric measures

Psychotomimetic side effects during ketamine infusion were assessed post hoc using the Altered States of Consciousness rating scale ‘5D-ASC’ [50]. The state-trait anxiety inventory (STAI X1) [51] and the Snaith–Hamilton Pleasure Scale (SHAPS) [52] were repeatedly used to assess subjective state and mood during the experiments (ratings before, 15 min, and 24 h after pharmacological intervention).

### fMRI data acquisition and analysis

Measurements were performed on a Philips Achieva TX 3-T whole-body MR unit equipped with an 8-channel head array. The subjects were told to lie still in the scanner with their eyes closed during the acquisition of resting state data. The functional images were collected in 10 min runs (200 volumes) using a sensitivity-encoded single-shot echo-planar sequence (TE = 35 ms; field of view = 22 cm; acquisition matrix = 80×80, interpolated to 128×128, 32 contiguous slices, voxelsize = 2.75×2.75×4 mm, and sensitivity-encoded acceleration factor R = 2.0) sensitive to BOLD contrast (T2* weighting). Using a midsagittal scout image, 32 contiguous axial slices were placed along the anterior-posterior commissure plane covering the entire brain and acquired with a repetition time of 3000 ms (θ = 82°) in ascending slice order. A 3-dimensional T1-weighted anatomical scan was obtained for structural reference.

Data were analyzed using the SPM8 (Wellcome Trust Center for Neuroimaging, London, England) based data processing assistant for resting state fMRI (DPARSF, Yan Chao-Gan, State Key Laboratory of Cognitive Neuroscience and Learning, Beijing Normal University, China [53]) which includes a rsfMRI data analysis toolkit (REST, by Song Xiao-Wei et al. [54]). The preprocessing steps followed the standard protocol described by Yan and Zang [53]. Functional data was corrected for differences in slice acquisition time, motion-corrected using a least squares approach and a six-parameter (rigid body) linear transformation, spatially normalized (to 3×3×3 mm isovoxels in standard space) and smoothed using a 4-mm full-width-at-half-maximum Gaussian kernel. The data was linearly detrended and filtered by a band pass filter (0.01–0.08 Hz) to suppress cardiac and respiratory motion induced effects. An additional regression of nuisance covariates was applied during which the functional data was corrected for the six head movement parameters and for global mean signal as well as for white matter and cerebrospinal fluid signal (defined according to Yan and Zang, 2010) [53].

### Seed region selection

**Figure 2:**
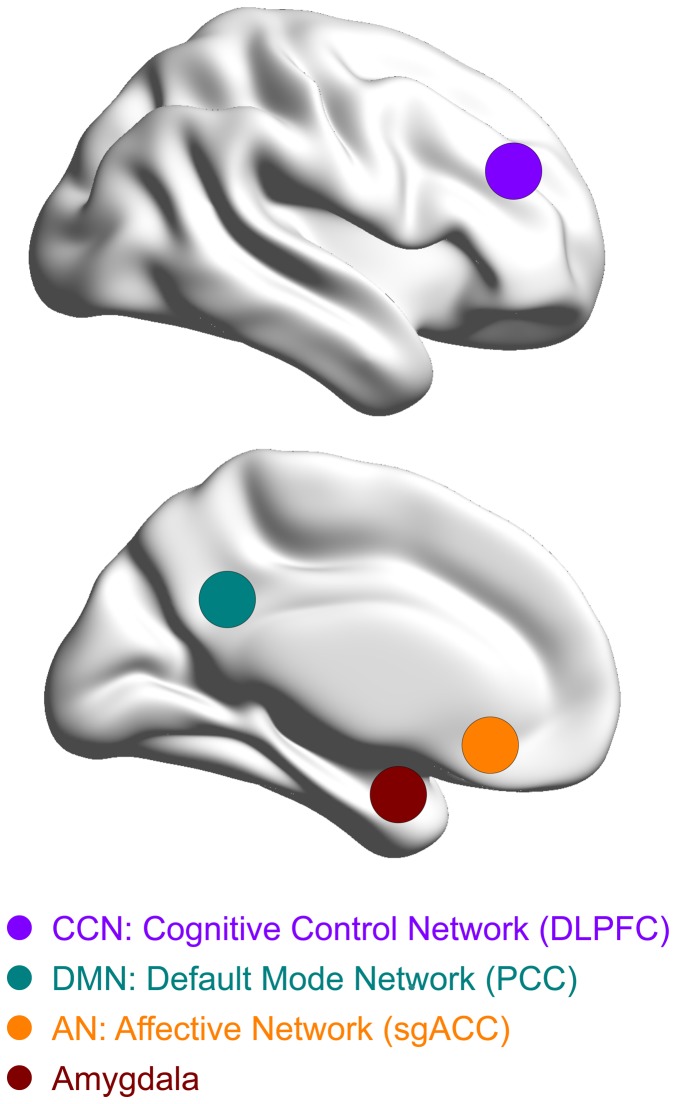
Seed region selection. Each of the four solid circles corresponds to a seed region in the Cognitive Control Network (CCN; purple): dorsolateral prefrontal cortex (DLPFC); in the Default Mode Network (DMN; green): posterior cingulate cortex (PCC); in the Affective Network (AN; orange): subgenual anterior cingulate cortex (sgACC); and in the amygdala (red).

We limited our analysis to a priori determined seed regions based on functional-anatomical network hypotheses and fMRI studies that yielded differential patterns of functional activation and connectivity in depressed patients and healthy subjects [21], [44], [46], [48]. Primarily, we focused on seed regions of interest (ROI: x, y, z, in Montreal Neurological Institute (MNI) space) in the cognitive control network (CCN), the default mode network (DMN), and affective networks (AN) that have been shown to exhibit increased resting state connectivity via the DN in depressed patients (s. Fig. 2): the left and right DLPFC (sphere at ±36 27 29 with 10 mm radius), the left and right PCC (sphere at ±6–50 24 with 7 mm radius), and the sgACC (sphere at 2 28–5 with 5 mm radius). Since in a recent study, decreased connectivity of the amygdala to prefrontal areas has been shown following antidepressant treatment [48], we further included anatomically defined ROIs for left and right amygdala, taken from the Automated Anatomical Labeling (AAL) atlas [55].

### Statistical analysis

Using the DPARSF toolbox, whole brain functional connectivity (FC) maps were obtained from the a priori determined seed regions of interest for each subject and every session separately. Statistical tests on regional functional connectivity maps were computed after application of Fisher's r-to-z transform, which yields variates that are approximately normally distributed. Paired t-test significance maps were computed in SPM8, based on the individual FC maps of the sessions baseline (ketamine) vs. follow-up (ketamine) and baseline (placebo) vs. follow-up (placebo). For the three seed-based comparisons, the correction of the t contrasts was made with a voxelwise threshold of p<0.001 and 15 voxels, achieving a corrected cluster threshold of p<0.05, as determined by the Monte Carlo simulations via AlphaSim (http://afni.nimh.nih.gov/afni) across whole brain (Gaussian filter width (FWHM) computed from the estimation of spatial smoothness of the residuals using AFNI (http://afni.nimh.nih.gov/afni): sigmax = 2.70, FWHMx = 6.36; sigmay = 2.70, FWHMy = 6.35; sigmaz = 2.78, FWHMz = 6.54; cluster connection radius (rmm) = 5.2 mm, individual voxel threshold probability = 0.001, 1000 iterations). Using the DPARSF toolbox, functional time series were extracted within each of the seed regions and in the target ROI of the dorsal nexus and in the MPFC/PACC voxel cluster, which was determined from the paired t-test significance maps. To quantify changes in functional connectivity of the seed regions to the target voxel clusters, correlation coefficients between the extracted functional time series were computed in Matlab R2009b (The MathWorks, Inc., Natick, MA, USA) for each subject and every session separately. Paired t-tests were applied to compare differences in Fisher z-transformed correlation values between baseline and follow-up sessions. Graphs of the mean changes in z-transformed correlation values were created using SigmaPlot (Systat Software Inc.). Whole brain paired t significance images were thresholded in SPM8 and visualized with the BrainNet Viewer (http://www.nitrc.org/projects/bnv/).

## Results

### Psychometric measures

Compared to placebo, subjects reported a significant increase in psychotomimetic symptoms following ketamine administration as assessed by the 5D-ASC questionnaire [50]. Ketamine treatment caused the most pronounced increase of scores in the scales of reduction of vigilance (n = 17, paired t-test: p<0.001), oceanic boundlesness (p = 0.005), anxious ego-dissolution (p<0.009), and visionary restructuralization (p<0.022). There was no significant correlation between psychotomimetic side effects and changes in functional connectivity of the AN und DMN seed regions to the DN. Ketamine or placebo treatment also did not affect subjective state and mood measured over the experimental period using the STAI X1 [51] and the Snaith-Hamilton Pleasure Scale (SHAPS) [52].

### Default mode network

**Figure 3:**
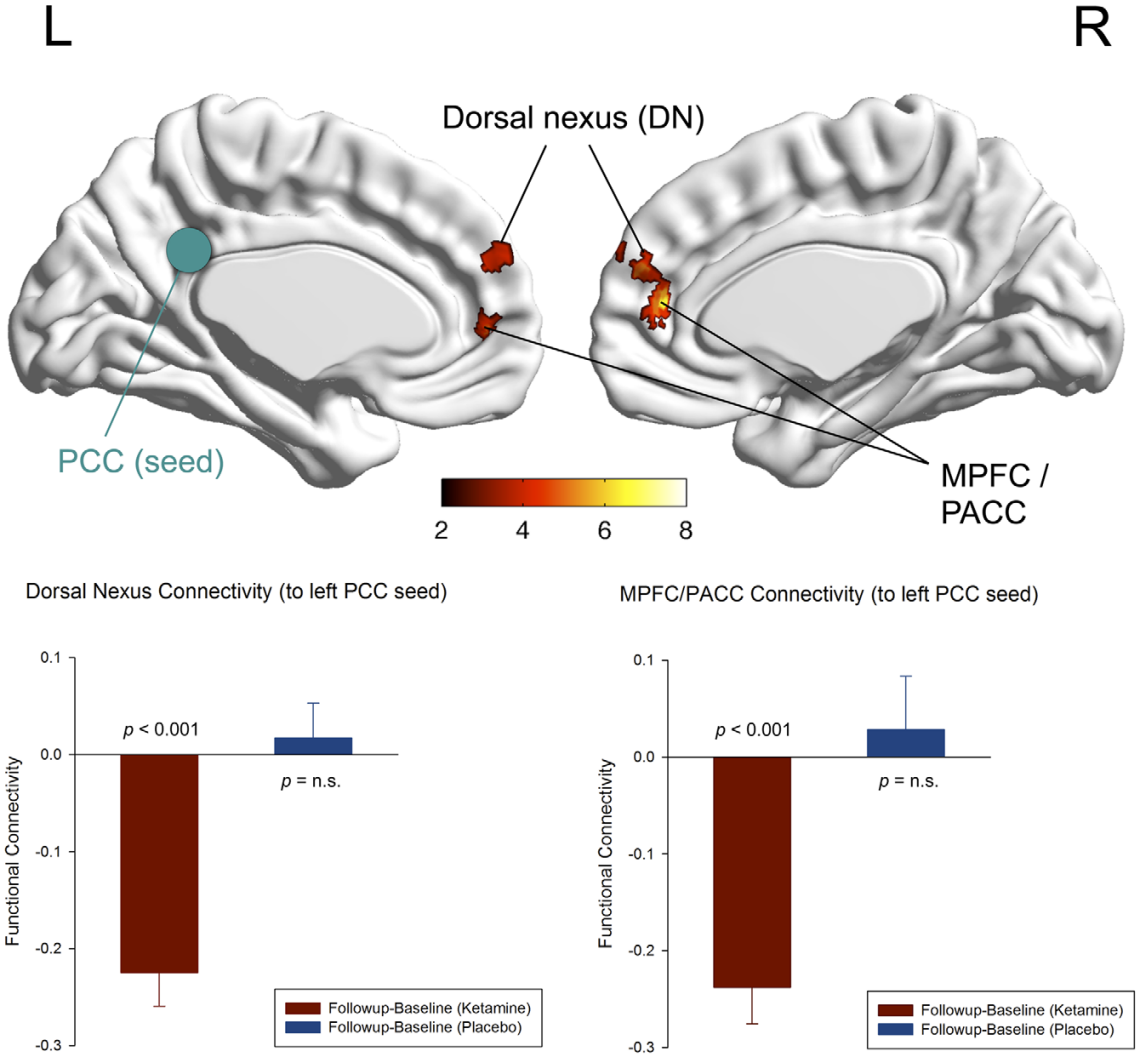
Functional connectivity of the default mode network (DMN). Significant voxels of the dorsal nexus (DMPFC) and pregenual anterior cingulate cortex (PACC) and medial prefrontal cortex (MPFC) showing reduced functional connectivity to the left posterior cingulate cortex (PCC) seed region (green) 24 hours after ketamine administration (n = 17, whole brain paired t-test: baseline(ketamine)-follow-up(ketamine); p_uncorr_<0.001, extent threshold of k>15, corresponds to a cluster-level p_corr_<0.05). The color bar indicates z values. The bar diagrams represent the change in functional connectivity (Fisher z-transformed correlation values) of the dorsal nexus (left) and the MPFC/PACC (right) to the left PCC from baseline to follow-up for the ketamine (red) and placebo condition (blue) (n = 17, paired t-test: p<0.001; error bars = s.e.m.).

At the whole brain level, we observed a focal decrease in functional connectivity between the left and right PCC seed region and the bilateral dorsal medial prefrontal cortex (DMPFC), the pregenual anterior cingulate (PACC) and the medioprefrontal cortex (MPFC) following ketamine administration (n = 17, paired t-test: p_uncorr_<0.001, extent threshold of k>15, resulting in a cluster-level p_corr_<0.05; Fig. 3). There were no other brain regions showing significant reductions in functional connectivity to the PCC seed regions. The difference in mean Fisher z-transformed correlation values extracted from the corresponding seed (PCC) and projection region in the bilateral DMPFC and MPFC/PACC was significant for the ketamine condition (n = 17, paired t-test (baseline-follow-up): p<0.001), with no change after placebo administration (Fig. 3 and 5).

### Affective network

**Figure 4:**
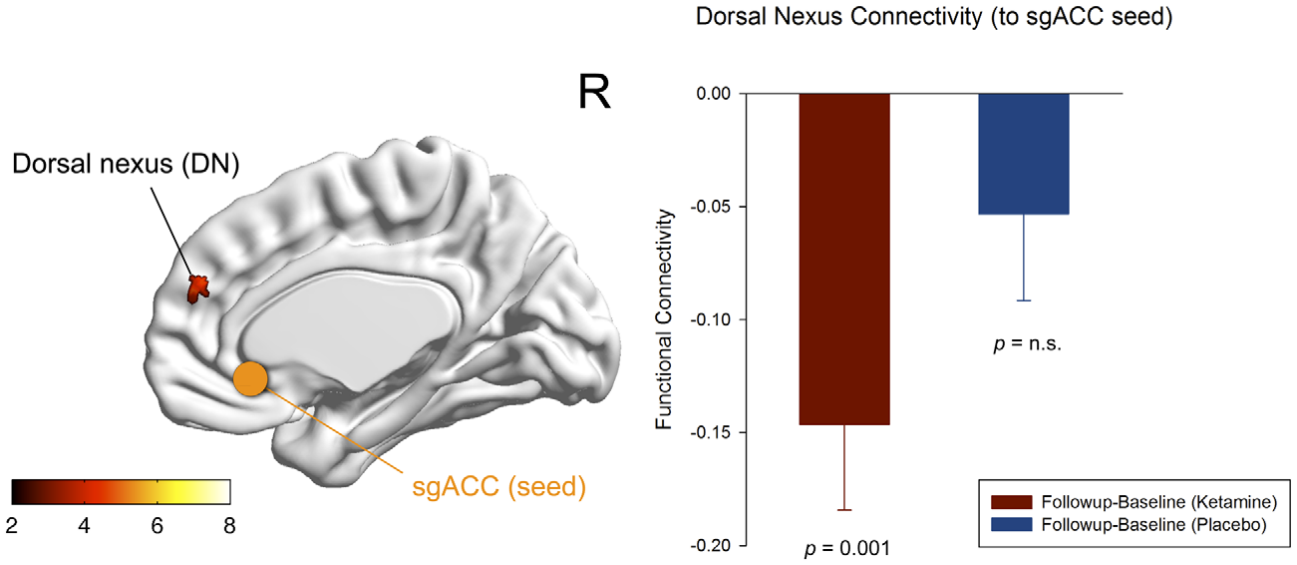
Functional connectivity of the affective network (AN). Significant voxels of the dorsal nexus (DMPFC) showing reduced functional connectivity to the subgenual anterior cingulate cortex (sgACC) seed region (blue) 24 hours after ketamine administration (n = 17, whole brain paired t-test: baseline(ketamine)-follow-up(ketamine); p_uncorr_<0.001, extent threshold of k>13, corresponds to a cluster-level p_corr_<0.1 indicating trend-level significance). The color bar indicates z values. The bar diagram represents the change in functional connectivity (Fisher z-transformed correlation values) of the dorsal nexus to the sgACC from baseline to follow-up for the ketamine (red) and placebo condition (blue) (n = 17, paired t-test: p = 0.001; error bars = s.e.m.).

**Figure 5:**
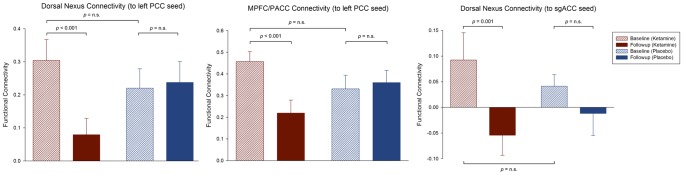
Functional connectivity across the whole experiment. The bar diagrams represent the functional connectivity (Fisher z-transformed correlation values after global mean regression) for the following experimental conditions: baseline and follow-up (ketamine; red); baseline and follow-up (placebo; blue). From left to right: Dorsal nexus (DN) connectivity to the left posterior cingulate cortex (PCC); medioprefrontal cortex (MPFC) and pregenual anterior cingulate cortex (PACC) connectivity to the left PCC; subgenual anterior cingulate cortex (sgACC) connectivity to the DN (n = 17, paired t-tests; error bars = s.e.m.).

At the whole brain level, we observed a statistical trend for a reduction in functional connectivity between the sgACC seed region and the right dorsal medial prefrontal cortex (DMPFC) following ketamine administration compared to placebo (n = 17, paired t-test: p_uncorr_<0.001, extent threshold of k>13, resulting in a cluster-level p_corr_<0.1; Fig. 4). The difference in mean Fisher z-transformed correlation values extracted from the corresponding seed (sgACC) and projection region (DMPFC) was significant for the ketamine condition (n = 17, paired t-test (baseline-follow-up): p = 0.001), with no significant change after placebo administration (Fig. 4 and 5).

### Other networks

No focal changes in functional connectivity with DLPFC were found in the whole brain paired t-test thresholded at p_uncorr_<0.001 (extent threshold of k>15, resulting in a cluster-level p_corr_<0.05) following ketamine administration. Likewise, no significant differences in functional connectivity between amygdala and prefrontal cortical areas could be observed after drug administration. Additional reductions in functional connectivity were found between amygdala and posterior parietal and premotor areas but the pattern was similar for both the drug and placebo condition.

### Whole brain dorsal nexus connectivity

In order to assess the specificity of our findings, we created an additional „dorsal nexus “seed (x: 6, y: 51, z: 24, volume: 513 mm^3^) based on the overlapping voxels that showed significant changes in functional connectivity to both the posterior and subgenual cingulate cortices after ketamine administration. At the whole brain level (using the same threshold as above), functional connectivity was reduced exclusively to the PCC but nowhere else in the brain.

## Discussion

While pharmacological effects of ketamine on task-induced fMRI BOLD signals have been studied extensively [56]–[62], this is the first randomized, placebo-controlled, double-blind, crossover study demonstrating changes in resting state functional connectivity in response to ketamine administration in healthy subjects. As our key finding we report a marked reduction of resting state functional connectivity between functional nodes of the default mode network (PCC) via the dorsal nexus (DN), pregenual anterior cingulate (PACC), and medioprefrontal cortex (MPFC) in healthy subjects 24 hours after ketamine administration compared to placebo. The term „dorsal nexus” was created recently by Sheline and colleagues (2010) to describe a functional node in the bilateral DMPFC with dramatically increased resting state connectivity to three important functional networks - the CCN, DMN, and AN - in patients suffering from major depression [46]. In our study, we aimed to model and identify ketamine-associated adaptations in healthy subjects within neural circuits that are relevant to the pathophysiology of MDD. Thus, the observed decrease in functional connectivity via the DN following ketamine administration in healthy subjects might have some implications for its therapeutic action in MDD patients. In light with the peak of ketamine's antidepressant effect 24 hours after intravenous administration [4], our findings suggest that this effect may be mediated by reducing the hyperconnectivity of the DN as shown here. Importantly, this action differs from previously reported effects of acute administration.

Antagonism at NMDA receptors has been shown to induce behavioral and neuroplastic changes in animal models relevant to certain aspects of the pathophysiology of depressive disorders [8]–[10]. The changes in resting state connectivity that we observed 24 hours post-infusion might thus result from adaptive changes in neuroglial glutamatergic throughput, neuroplasticity and information processing in specific neurocircuits. In strong support for such a glutamatergic mechanism of action a recent study reported a direct relationship between aberrant resting state functional connectivities and glutamatergic imbalance in depressed patients across distinct functional networks [35]. This supports our hypothesis that glutamatergic modulation by specific drugs like ketamine exerts its antidepressant effects via reconfiguration of resting state functional connectivity.

**Figure 6:**
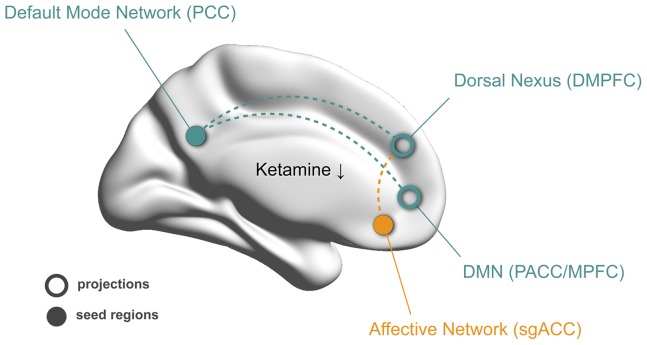
Proposed hypothetical model of ketamine-associated changes in functional connectivity. In the healthy human brain, a single antidepressant dose of ketamine reduces functional connectivity of the dorsal nexus (DN) to the Default Mode Network (DMN; green). The reduction in functional connectivity of the Affective Network (AN; orange) to the DN reached trend-level significance only (s. Fig. 4), possibly due to the absence of any pre-existing hyperconnectivity in healthy subjects. This action may serve as a model for the discovery of novel antidepressant biomechanisms in major depression where functional connectivity of the DMN and AN via the DN is increased. The solid circles correspond to seed regions in the posterior cingulate cortex (PCC; green) and subgenual anterior cingulate cortex (sgACC; orange). The correspondingly colored open circles and dotted lines represent regions with decreased connectivity with the respective seed regions after ketamine administration. This hypothetical model is based on results from previous rsfMRI studies in MDD patients and on data obtained in healthy subjects after ketamine administration and needs to be further verified in MDD patients receiving ketamine.

The psychophysiological relevance of reducing functional hyperconnectivities within and between resting state networks like the DMN or the AN is given by their involvement in circumscribed aspects of the depressive psychopathology. Regions of the DMN commonly show the greatest activity at rest and decrease their level of activity during goal-directed tasks [37] and are thought to be involved in self-referential processes such as introspection, remembering, and planning [63]. In patients with major depression, a failure to normally down-regulate activity within the DMN during external stimulation was found [38], [39], with increasing levels of DMN dominance being associated with higher levels of maladaptive, depressive rumination and lower levels of adaptive, reflective rumination [64]. Thus, the reduction in functional connectivity between anterior (PACC/MPFC) and posterior parts of the DMN (PCC) that we observed after ketamine administration in healthy subjects may have implications for antidepressant treatment in terms of a reduction of the increased level of DMN dominance (s. Fig. 6).

Moreover, the sgACC as a critical hub of the AN plays an important role in emotion processing and the pathogenesis of mood disorders and has become a promising target for deep brain stimulation in patients with severe, refractory depression [65]. A number of structural, metabolic and functional abnormalities has been identified in the sgACC of MDD patients [66]. Resting state sgACC functional connectivity with the DMN was significantly greater in depressed subjects and correlated positively with the length of the current depressive episode [44]. As proposed by Sheline et al. (2010) an attentional shift with increased self-focus might interfere with task performance in the CCN through increased resting state DMN connectivity with the DN [46]. The hot-wiring of the sgACC to those systems might further explain its maladaptive contribution to negative self-monitoring and reduced task-performance in MDD, given its role in the regulation of visceral functions and sad mood [66]. Compared to the reduction in DMN to DN connectivity after ketamine administration in healthy subjects, the reduction of AN to DN connectivity was less pronounced reaching statistical trend-level only and has therefore to be considered preliminary. The absence of a pre-existing hyperconnectivity of the sgACC to the DN in healthy subjects might explain the limited dynamic range in terms of a reduction in functional connectivity in our study, while this mechanism may become relevant in a clinical population (s. Fig. 6).

Our findings suggest that intravenous ketamine in healthy subjects affects primarily the DMN (PCC) connectivity via the DN and PACC/MPFC one day after infusion. We could not find any focal change in connectivity to the CCN following ketamine administration and contrary to resting state studies with serotonergic and noradrenergic antidepressants including citalopram and reboxetine [48], functional connectivity of the prefrontal cortex to the amygdala remained unaffected by ketamine. Hence, the circumscribed effect of ketamine on DMN connectivity to the DN supports the hypothesis that effective antidepressant treatment involves systematic alterations in connections among higher-order functional networks via nodes such as the DN. However, those putative implications for MDD have to be regarded as preliminary since the results reported here are based on healthy subjects. Apart from this limitation, our aim of addressing systems level mechanisms of ketamine's antidepressant action is reflected in our elaborate study design including a 24 h post-infusion interval, appropriate dosage and duration of the ketamine infusion, and the selection of seed regions that are relevant to MDD. Therefore, our findings may serve as a model to elucidate potential biomechanisms of drug action in the absence of any pre-existing homeostatic dysregulation as part of the disease process, medication status, or comorbidity. In a next step, the explanatory power of our observation has to be further confirmed in a randomized-controlled clinical trial in MDD patients receiving ketamine. Moreover, our results do not allow any conclusions to be drawn for the action of ketamine on the healthy human brain in general or in the context of ketamine as a model for schizophrenia.

In conclusion, we report a reduction of functional connectivity in networks that play a critical role in the pathophysiology of MDD in healthy subjects 24 hours after receiving an antidepressant dose of ketamine. Based on those findings we raise the hypothesis that reducing functional connectivity of the dorsal nexus reflects underlying molecular mechanisms relevant to the antidepressant efficacy of ketamine. Whether this circuit-level glutamatergic effect is likely to be associated with reversing aspects of emotional and behavioral dysregulation has to be further investigated in a clinical study involving MDD patients. This is in further support of the notion of using ketamine as a research tool into the neurobiology of mood disorders and to delineate potential biomarkers and action mechanisms of antidepressant treatment response.
